# A Shakespearian Medical Question

**Published:** 1885-12

**Authors:** Ad. Lippe

**Affiliations:** Philadelphia


### HOMŒOPATHIC PHYSICIAN,

A MONTHLY JOURNAL OF MEDICAL SCIENCE.

"If our school ever gives up the strict inductive method of Hahnemann, we are lost, and deserve only to be mentioned as a caricature in the history of medicine."—CONSTANTINE HERING.

Vol. V. DECEMBER, 1885. No. 12.

### A SHAKESPEARIAN MEDICAL QUESTION.

AD. LIPPE, M. D., PHILADELPHIA.

The Shakespearians have been discussing the possibility of having at last found out Shakespeare in a mistake. The passage over which the question arises is to be found in *Othello*, last act, and reads—

> "Now, how dost thou look now? O ill starr'd wench!
> Pale as thy smock!"

Some Shakespearians believed it to be an error committed by Shakespeare to have Desdemona look pale after Othello had smothered her; that more likely her face appeared bloated and blackened. It is evident that Othello *smothered* Desdemona, but he did just as evidently not strangle her; she died from *suffocation*. A strangled person appears bloated, the countenance is suffused, the marks of strangulation are visible on the neck and show how the circulation was forcibly interrupted, causing the clotting of the blood in the carotid arteries; the tongue and eyes are protruding. In Wharton and Stillé's *Medical Jurisprudence*, Vol. II, p. 802, we find a complete vindication of Shakespeare. Paragraph 902, IV. "*Homicidal Suffocation.* Those who are usually the victims of this form of murder are infants and the aged, or those who are otherwise helpless. So slight a degree of resistance is necessary to defeat the purpose of the assassin that a great disproportion of strength must exist for the attempt to be successful. Nevertheless, those miserable wretches, Burke and his accomplices, reduced murder by suffocation to a system, choosing it as a mode of death most likely to leave no mark of crime behind it. The murderer bore with his whole weight upon the breast of his victim and with his hands covered forcibly the mouth and nostrils till death came on. The body of one of the victims presented, according to Dr. Christison, so few traces of injury, that, without the assistance of proof from other sources, it would have been impossible to have declared that the death was not a natural one."

How well Shakespeare knew the different appearance of a suffocated and a strangled person is evidenced in his masterly description of strangulation in Part II of *Henry VI*, Act iii, Scene 2.* Suffocation alone caused the death of Desdemona, and suffocation was slow. Othello, supposing Desdemona dead, admits Emilia to the chamber, who relates to him the killing of Roderigo, whereupon Desdemona speaks out, "O falsely, falsely murdered!" and when Emilia begs her sweet mistress to speak again, Desdemona tells her, "A guiltless death I die!" and when Emilia asks her, "Oh! who hath done this deed?" Desdemona replies, "Nobody; I myself. Farewell: commend me to my kind Lord; oh! farewell!" and dies. This last gasp of breath is explainable on the supposition that suffocation, almost accomplished, left some of the vessels of the lungs still free and a very small quantity of blood in the left side of the heart. Violent mental emotions caused the heart to contract, and when the right side of the heart finally became filled with dark venous blood she died.

---

* *Warwick:* "See how the blood is settled in his face!
Oft have I seen a timely parted ghost,
Of ashy semblance, meagre, pale, and bloodless,
Being all descended to the laboring heart,
Who in the conflict that it holds with death,
Attracts the same for aidance 'gainst the enemy,
Which with the heart there cools and ne'er returneth
To blush and beautify the cheek again.
But, see, his face is black and full of blood;
His eye-balls further out than when he liv'd;
Staring full ghastly like a strangled man;
His hair upreared, his nostrils stretch'd with struggling;
His hands abroad display'd, as one that grasp'd
And tugg'd for life, and was by strength subdued:
Look at the sheets; his hair, you see, is sticking;
His well proportioned beard made rough and rugged,
Like to the summer's corn by tempest lodg'd.
It cannot be but he was murder'd here;
The least of all these signs were probable."

COMMENTS.—This reflection is to show the great knowledge the poet had of all things; how most accurately he draws the distinction between strangulation and suffocation in his description of the appearances after death. His descriptions are unsurpassed by anything modern sciences have revealed. On further reflection it appears that every medical man should be fully versed in medical jurisprudence, as cases will come up in which such a knowledge of one of the many branches of medical science—in this case a knowledge of medical jurisprudence—is absolutely needed.

